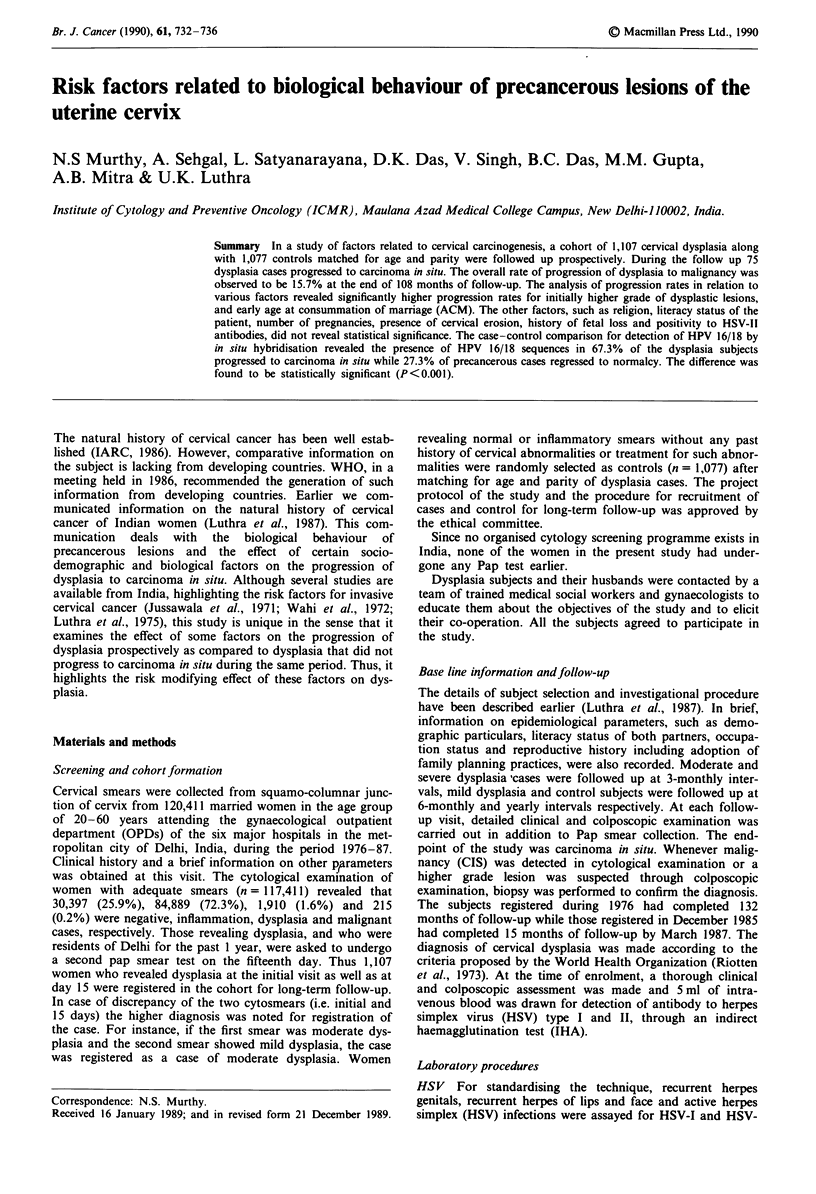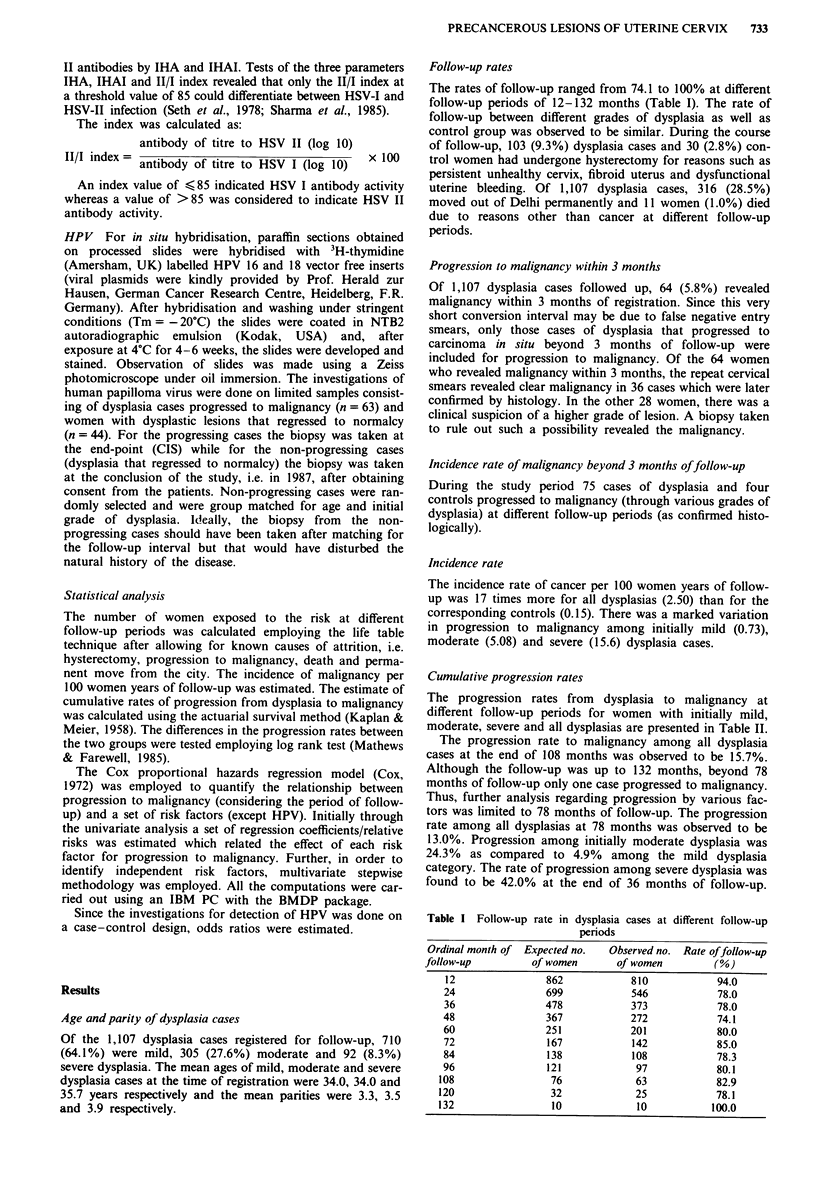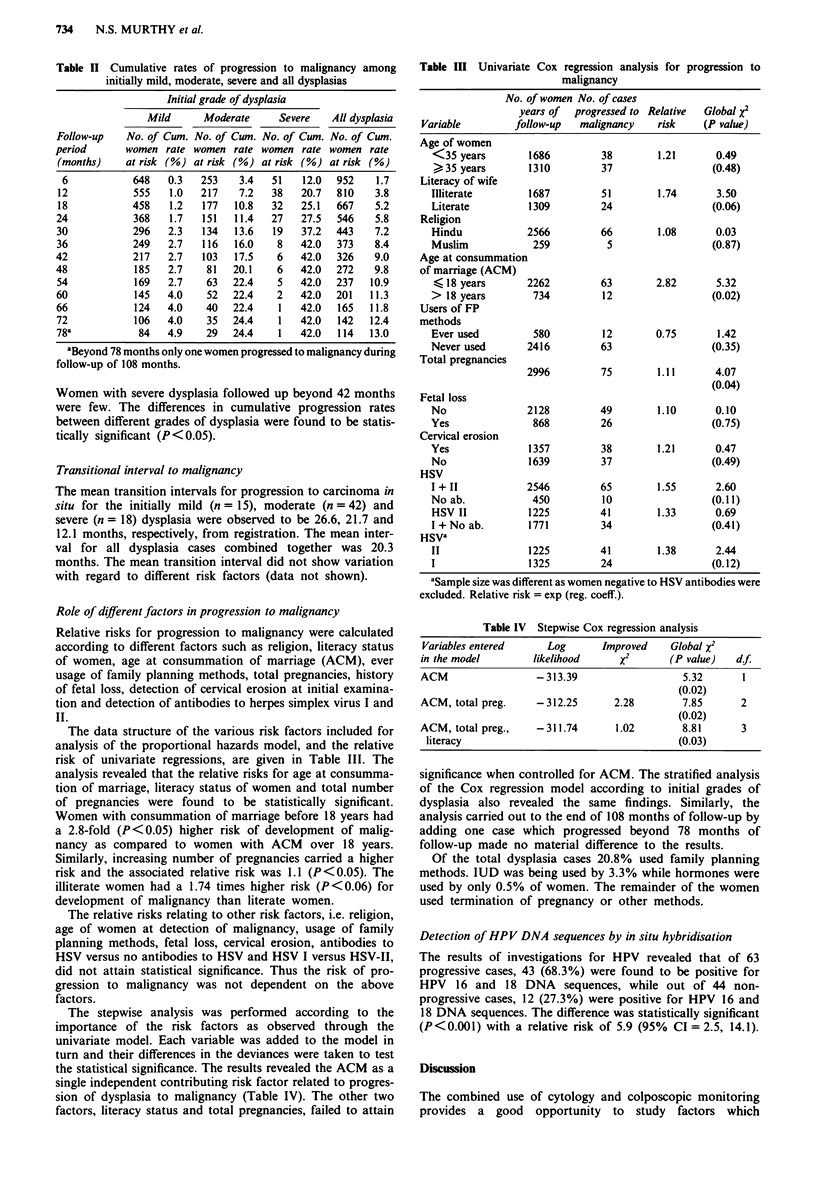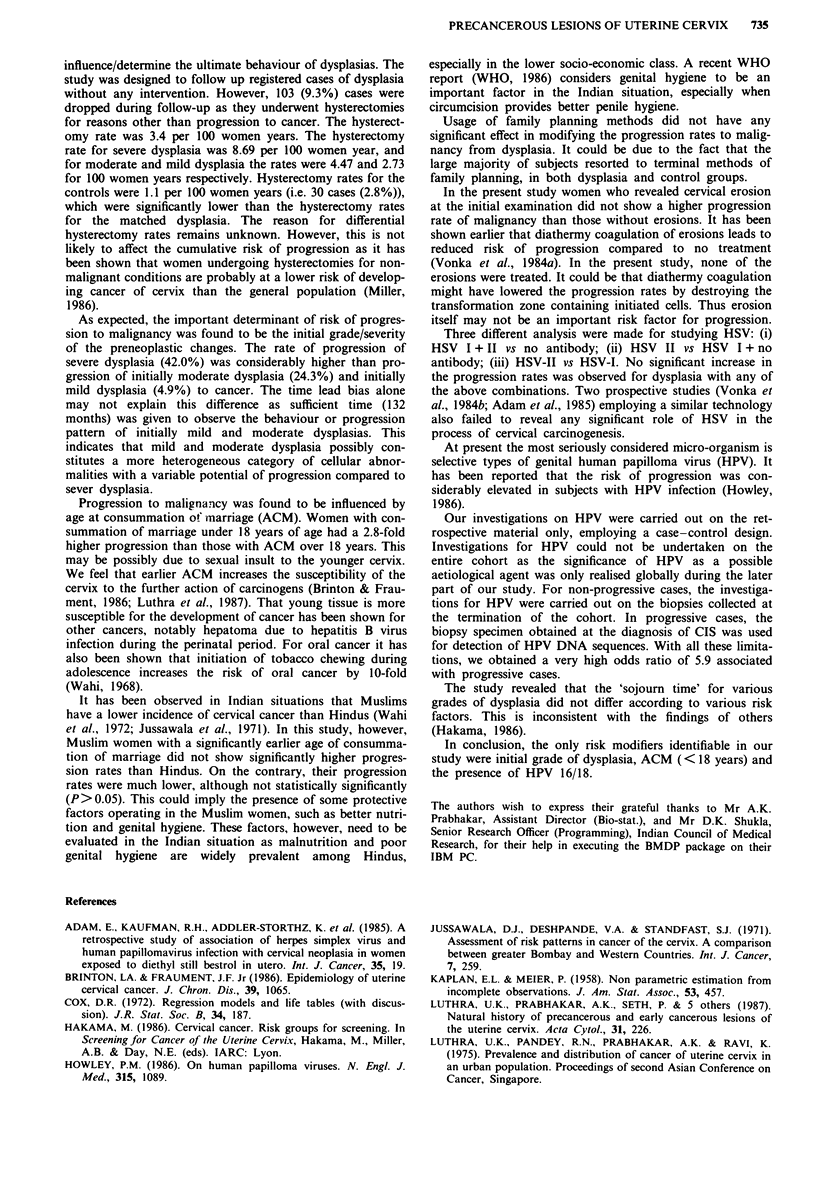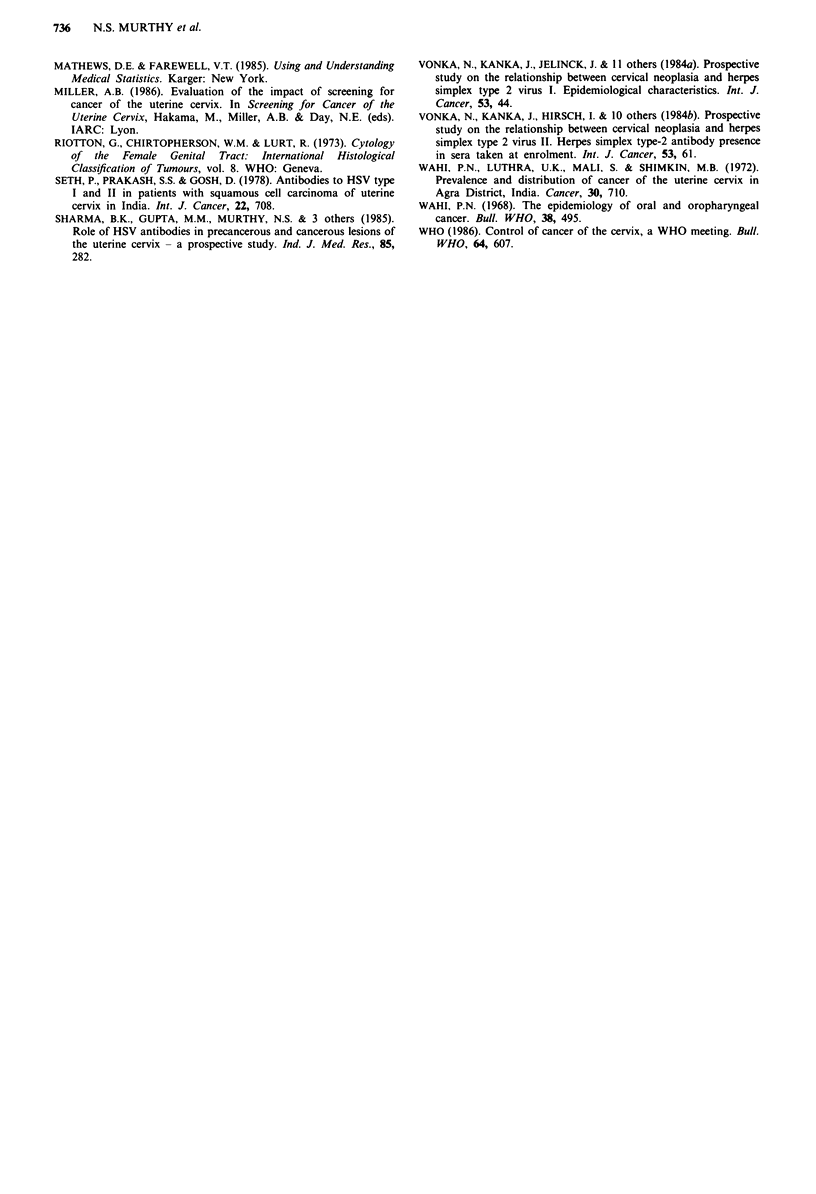# Risk factors related to biological behaviour of precancerous lesions of the uterine cervix.

**DOI:** 10.1038/bjc.1990.164

**Published:** 1990-05

**Authors:** N. S. Murthy, A. Sehgal, L. Satyanarayana, D. K. Das, V. Singh, B. C. Das, M. M. Gupta, A. B. Mitra, U. K. Luthra

**Affiliations:** Institute of Cytology and Preventive Oncology (ICMR), Maulana Azad Medical College Campus, New Delhi, India.

## Abstract

In a study of factors related to cervical carcinogenesis, a cohort of 1,107 cervical dysplasia along with 1,077 controls matched for age and parity were followed up prospectively. During the follow up 75 dysplasia cases progressed to carcinoma in situ. The overall rate of progression of dysplasia to malignancy was observed to be 15.7% at the end of 108 months of follow-up. The analysis of progression rates in relation to various factors revealed significantly higher progression rates for initially higher grade of dysplastic lesions, and early age at consummation of marriage (ACM). The other factors, such as religion, literacy status of the patient, number of pregnancies, presence of cervical erosion, history of fetal loss and positivity to HSV-II antibodies, did not reveal statistical significance. The case-control comparison for detection of HPV 16/18 by in situ hybridisation revealed the presence of HPV 16/18 sequences in 67.3% of the dysplasia subjects progressed to carcinoma in situ while 27.3% of precancerous cases regressed to normalcy. The difference was found to be statistically significant (P less than 0.001).


*Br. J. Cancer* (1990), **61**, 732–736 © Macmillan Press Ltd., 1990

### Risk factors related to biological behaviour of precancerous lesions of the uterine cervix

N.S Murthy, A. Sehgal, L. Satyanarayana, D.K. Das, V. Singh, B.C. Das, M.M. Gupta, A.B. Mitra & U.K. Luthra

*Institute of Cytology and Preventive Oncology (ICMR), Maulana Azad Medical College Campus, New Delhi-110002, India.*

**Summary** In a study of factors related to cervical carcinogenesis, a cohort of 1,107 cervical dysplasia along with 1,077 controls matched for age and parity were followed up prospectively. During the follow up 75 dysplasia cases progressed to carcinoma *in situ*. The overall rate of progression of dysplasia to malignancy was observed to be 15.7% at the end of 108 months of follow-up. The analysis of progression rates in relation to various factors revealed significantly higher progression rates for initially higher grade of dysplastic lesions, and early age at consummation of marriage (ACM). The other factors, such as religion, literacy status of the patient, number of pregnancies, presence of cervical erosion, history of fetal loss and positivity to HSV-II antibodies, did not reveal statistical significance. The case-control comparison for detection of HPV 16/18 by *in situ* hybridisation revealed the presence of HPV 16/18 sequences in 67.3% of the dysplasia subjects progressed to carcinoma *in situ* while 27.3% of precancerous cases regressed to normalcy. The difference was found to be statistically significant ($P<0.001$).

The natural history of cervical cancer has been well established (IARC, 1986). However, comparative information on the subject is lacking from developing countries. WHO, in a meeting held in 1986, recommended the generation of such information from developing countries. Earlier we communicated information on the natural history of cervical cancer of Indian women (Luthra *et al.*, 1987). This communication deals with the biological behaviour of precancerous lesions and the effect of certain socio-demographic and biological factors on the progression of dysplasia to carcinoma *in situ*. Although several studies are available from India, highlighting the risk factors for invasive cervical cancer (Jussawala *et al.*, 1971; Wahi *et al.*, 1972; Luthra *et al.*, 1975), this study is unique in the sense that it examines the effect of some factors on the progression of dysplasia prospectively as compared to dysplasia that did not progress to carcinoma *in situ* during the same period. Thus, it highlights the risk modifying effect of these factors on dysplasia.

### Materials and methods

### *Screening and cohort formation*

Cervical smears were collected from squamo-columnar junction of cervix from 120,411 married women in the age group of 20–60 years attending the gynaecological outpatient department (OPDs) of the six major hospitals in the metropolitan city of Delhi, India, during the period 1976–87. Clinical history and a brief information on other parameters was obtained at this visit. The cytological examination of women with adequate smears ($n = 117{,}411$) revealed that 30,397 (25.9%), 84,889 (72.3%), 1,910 (1.6%) and 215 (0.2%) were negative, inflammation, dysplasia and malignant cases, respectively. Those revealing dysplasia, and who were residents of Delhi for the past 1 year, were asked to undergo a second pap smear test on the fifteenth day. Thus 1,107 women who revealed dysplasia at the initial visit as well as at day 15 were registered in the cohort for long-term follow-up. In case of discrepancy of the two cytosmears (i.e. initial and 15 days) the higher diagnosis was noted for registration of the case. For instance, if the first smear was moderate dysplasia and the second smear showed mild dysplasia, the case was registered as a case of moderate dysplasia. Women revealing normal or inflammatory smears without any past history of cervical abnormalities or treatment for such abnormalities were randomly selected as controls ($n = 1{,}077$) after matching for age and parity of dysplasia cases. The project protocol of the study and the procedure for recruitment of cases and control for long-term follow-up was approved by the ethical committee.

Since no organised cytology screening programme exists in India, none of the women in the present study had undergone any Pap test earlier.

Dysplasia subjects and their husbands were contacted by a team of trained medical social workers and gynaecologists to educate them about the objectives of the study and to elicit their co-operation. All the subjects agreed to participate in the study.

### *Base line information and follow-up*

The details of subject selection and investigational procedure have been described earlier (Luthra *et al.*, 1987). In brief, information on epidemiological parameters, such as demographic particulars, literacy status of both partners, occupation status and reproductive history including adoption of family planning practices, were also recorded. Moderate and severe dysplasia cases were followed up at 3-monthly intervals, mild dysplasia and control subjects were followed up at 6-monthly and yearly intervals respectively. At each follow-up visit, detailed clinical and colposcopic examination was carried out in addition to Pap smear collection. The end-point of the study was carcinoma *in situ*. Whenever malignancy (CIS) was detected in cytological examination or a higher grade lesion was suspected through colposcopic examination, biopsy was performed to confirm the diagnosis. The subjects registered during 1976 had completed 132 months of follow-up while those registered in December 1985 had completed 15 months of follow-up by March 1987. The diagnosis of cervical dysplasia was made according to the criteria proposed by the World Health Organization (Riotten *et al.*, 1973). At the time of enrolment, a thorough clinical and colposcopic assessment was made and 5 ml of intravenous blood was drawn for detection of antibody to herpes simplex virus (HSV) type I and II, through an indirect haemagglutination test (IHA).

### *Laboratory procedures*

*HSV* For standardising the technique, recurrent herpes genitals, recurrent herpes of lips and face and active herpes simplex (HSV) infections were assayed for HSV-I and HSV-

Correspondence: N.S. Murthy.
Received 16 January 1989; and in revised form 21 December 1989.

II antibodies by IHA and IHAI. Tests of the three parameters IHA, IHAI and II/I index revealed that only the II/I index at a threshold value of 85 could differentiate between HSV-I and HSV-II infection (Seth *et al.*, 1978; Sharma *et al.*, 1985).

The index was calculated as:

$$\text{II/I index} = \frac{\text{antibody of titre to HSV II (log 10)}}{\text{antibody of titre to HSV I (log 10)}} \times 100$$

An index value of $\leqslant 85$ indicated HSV I antibody activity whereas a value of $>85$ was considered to indicate HSV II antibody activity.

*HPV* For *in situ* hybridisation, paraffin sections obtained on processed slides were hybridised with $^3$H-thymidine (Amersham, UK) labelled HPV 16 and 18 vector free inserts (viral plasmids were kindly provided by Prof. Herald zur Hausen, German Cancer Research Centre, Heidelberg, F.R. Germany). After hybridisation and washing under stringent conditions ($Tm = -20°C$) the slides were coated in NTB2 autoradiographic emulsion (Kodak, USA) and, after exposure at 4°C for 4–6 weeks, the slides were developed and stained. Observation of slides was made using a Zeiss photomicroscope under oil immersion. The investigations of human papilloma virus were done on limited samples consisting of dysplasia cases progressed to malignancy ($n = 63$) and women with dysplastic lesions that regressed to normalcy ($n = 44$). For the progressing cases the biopsy was taken at the end-point (CIS) while for the non-progressing cases (dysplasia that regressed to normalcy) the biopsy was taken at the conclusion of the study, i.e. in 1987, after obtaining consent from the patients. Non-progressing cases were randomly selected and were group matched for age and initial grade of dysplasia. Ideally, the biopsy from the non-progressing cases should have been taken after matching for the follow-up interval but that would have disturbed the natural history of the disease.

### *Statistical analysis*

The number of women exposed to the risk at different follow-up periods was calculated employing the life table technique after allowing for known causes of attrition, i.e. hysterectomy, progression to malignancy, death and permanent move from the city. The incidence of malignancy per 100 women years of follow-up was estimated. The estimate of cumulative rates of progression from dysplasia to malignancy was calculated using the actuarial survival method (Kaplan & Meier, 1958). The differences in the progression rates between the two groups were tested employing log rank test (Mathews & Farewell, 1985).

The Cox proportional hazards regression model (Cox, 1972) was employed to quantify the relationship between progression to malignancy (considering the period of follow-up) and a set of risk factors (except HPV). Initially through the univariate analysis a set of regression coefficients/relative risks was estimated which related the effect of each risk factor for progression to malignancy. Further, in order to identify independent risk factors, multivariate stepwise methodology was employed. All the computations were carried out using an IBM PC with the BMDP package.

Since the investigations for detection of HPV was done on a case–control design, odds ratios were estimated.

### Results

### *Age and parity of dysplasia cases*

Of the 1,107 dysplasia cases registered for follow-up, 710 (64.1%) were mild, 305 (27.6%) moderate and 92 (8.3%) severe dysplasia. The mean ages of mild, moderate and severe dysplasia cases at the time of registration were 34.0, 34.0 and 35.7 years respectively and the mean parities were 3.3, 3.5 and 3.9 respectively.

### *Follow-up rates*

The rates of follow-up ranged from 74.1 to 100% at different follow-up periods of 12–132 months (Table I). The rate of follow-up between different grades of dysplasia as well as control group was observed to be similar. During the course of follow-up, 103 (9.3%) dysplasia cases and 30 (2.8%) control women had undergone hysterectomy for reasons such as persistent unhealthy cervix, fibroid uterus and dysfunctional uterine bleeding. Of 1,107 dysplasia cases, 316 (28.5%) moved out of Delhi permanently and 11 women (1.0%) died due to reasons other than cancer at different follow-up periods.

### *Progression to malignancy within 3 months*

Of 1,107 dysplasia cases followed up, 64 (5.8%) revealed malignancy within 3 months of registration. Since this very short conversion interval may be due to false negative entry smears, only those cases of dysplasia that progressed to carcinoma *in situ* beyond 3 months of follow-up were included for progression to malignancy. Of the 64 women who revealed malignancy within 3 months, the repeat cervical smears revealed clear malignancy in 36 cases which were later confirmed by histology. In the other 28 women, there was a clinical suspicion of a higher grade of lesion. A biopsy taken to rule out such a possibility revealed the malignancy.

### *Incidence rate of malignancy beyond 3 months of follow-up*

During the study period 75 cases of dysplasia and four controls progressed to malignancy (through various grades of dysplasia) at different follow-up periods (as confirmed histologically).

### *Incidence rate*

The incidence rate of cancer per 100 women years of follow-up was 17 times more for all dysplasias (2.50) than for the corresponding controls (0.15). There was a marked variation in progression to malignancy among initially mild (0.73), moderate (5.08) and severe (15.6) dysplasia cases.

### *Cumulative progression rates*

The progression rates from dysplasia to malignancy at different follow-up periods for women with initially mild, moderate, severe and all dysplasias are presented in Table II.

The progression rate to malignancy among all dysplasia cases at the end of 108 months was observed to be 15.7%. Although the follow-up was up to 132 months, beyond 78 months of follow-up only one case progressed to malignancy. Thus, further analysis regarding progression by various factors was limited to 78 months of follow-up. The progression rate among all dysplasias at 78 months was observed to be 13.0%. Progression among initially moderate dysplasia was 24.3% as compared to 4.9% among the mild dysplasia category. The rate of progression among severe dysplasia was found to be 42.0% at the end of 36 months of follow-up.

**Table I** Follow-up rate in dysplasia cases at different follow-up periods

| *Ordinal month of follow-up* | *Expected no. of women* | *Observed no. of women* | *Rate of follow-up (%)* |
|---|---|---|---|
| 12 | 862 | 810 | 94.0 |
| 24 | 699 | 546 | 78.0 |
| 36 | 478 | 373 | 78.0 |
| 48 | 367 | 272 | 74.1 |
| 60 | 251 | 201 | 80.0 |
| 72 | 167 | 142 | 85.0 |
| 84 | 138 | 108 | 78.3 |
| 96 | 121 | 97 | 80.1 |
| 108 | 76 | 63 | 82.9 |
| 120 | 32 | 25 | 78.1 |
| 132 | 10 | 10 | 100.0 |

**Table II** Cumulative rates of progression to malignancy among initially mild, moderate, severe and all dysplasias

| | *Initial grade of dysplasia* | | | | | | | |
|---|---|---|---|---|---|---|---|---|
| | *Mild* | | *Moderate* | | *Severe* | | *All dysplasia* | |
| *Follow-up period (months)* | *No. of women at risk* | *Cum. rate (%)* | *No. of women at risk* | *Cum. rate (%)* | *No. of women at risk* | *Cum. rate (%)* | *No. of women at risk* | *Cum. rate (%)* |
| 6 | 648 | 0.3 | 253 | 3.4 | 51 | 12.0 | 952 | 1.7 |
| 12 | 555 | 1.0 | 217 | 7.2 | 38 | 20.7 | 810 | 3.8 |
| 18 | 458 | 1.2 | 177 | 10.8 | 32 | 25.1 | 667 | 5.2 |
| 24 | 368 | 1.7 | 151 | 11.4 | 27 | 27.5 | 546 | 5.8 |
| 30 | 296 | 2.3 | 134 | 13.6 | 19 | 37.2 | 443 | 7.2 |
| 36 | 249 | 2.7 | 116 | 16.0 | 8 | 42.0 | 373 | 8.4 |
| 42 | 217 | 2.7 | 103 | 17.5 | 6 | 42.0 | 326 | 9.0 |
| 48 | 185 | 2.7 | 81 | 20.1 | 6 | 42.0 | 272 | 9.8 |
| 54 | 169 | 2.7 | 63 | 22.4 | 5 | 42.0 | 237 | 10.9 |
| 60 | 145 | 4.0 | 52 | 22.4 | 2 | 42.0 | 201 | 11.3 |
| 66 | 124 | 4.0 | 40 | 22.4 | 1 | 42.0 | 165 | 11.8 |
| 72 | 106 | 4.0 | 35 | 24.4 | 1 | 42.0 | 142 | 12.4 |
| 78[a] | 84 | 4.9 | 29 | 24.4 | 1 | 42.0 | 114 | 13.0 |

[a]Beyond 78 months only one women progressed to malignancy during follow-up of 108 months.

Women with severe dysplasia followed up beyond 42 months were few. The differences in cumulative progression rates between different grades of dysplasia were found to be statistically significant ($P<0.05$).

### *Transitional interval to malignancy*

The mean transition intervals for progression to carcinoma *in situ* for the initially mild ($n = 15$), moderate ($n = 42$) and severe ($n = 18$) dysplasia were observed to be 26.6, 21.7 and 12.1 months, respectively, from registration. The mean interval for all dysplasia cases combined together was 20.3 months. The mean transition interval did not show variation with regard to different risk factors (data not shown).

### *Role of different factors in progression to malignancy*

Relative risks for progression to malignancy were calculated according to different factors such as religion, literacy status of women, age at consummation of marriage (ACM), ever usage of family planning methods, total pregnancies, history of fetal loss, detection of cervical erosion at initial examination and detection of antibodies to herpes simplex virus I and II.

The data structure of the various risk factors included for analysis of the proportional hazards model, and the relative risk of univariate regressions, are given in Table III. The analysis revealed that the relative risks for age at consummation of marriage, literacy status of women and total number of pregnancies were found to be statistically significant. Women with consummation of marriage before 18 years had a 2.8-fold ($P<0.05$) higher risk of development of malignancy as compared to women with ACM over 18 years. Similarly, increasing number of pregnancies carried a higher risk and the associated relative risk was 1.1 ($P<0.05$). The illiterate women had a 1.74 times higher risk ($P<0.06$) for development of malignancy than literate women.

The relative risks relating to other risk factors, i.e. religion, age of women at detection of malignancy, usage of family planning methods, fetal loss, cervical erosion, antibodies to HSV versus no antibodies to HSV and HSV I versus HSV-II, did not attain statistical significance. Thus the risk of progression to malignancy was not dependent on the above factors.

The stepwise analysis was performed according to the importance of the risk factors as observed through the univariate model. Each variable was added to the model in turn and their differences in the deviances were taken to test the statistical significance. The results revealed the ACM as a single independent contributing risk factor related to progression of dysplasia to malignancy (Table IV). The other two factors, literacy status and total pregnancies, failed to attain significance when controlled for ACM. The stratified analysis of the Cox regression model according to initial grades of dysplasia also revealed the same findings. Similarly, the analysis carried out to the end of 108 months of follow-up by adding one case which progressed beyond 78 months of follow-up made no material difference to the results.

Of the total dysplasia cases 20.8% used family planning methods. IUD was being used by 3.3% while hormones were used by only 0.5% of women. The remainder of the women used termination of pregnancy or other methods.

**Table III** Univariate Cox regression analysis for progression to malignancy

| *Variable* | *No. of women years of follow-up* | *No. of cases progressed to malignancy* | *Relative risk* | *Global $\chi^2$ (P value)* |
|---|---|---|---|---|
| Age of women | | | | |
| <35 years | 1686 | 38 | 1.21 | 0.49 |
| ⩾35 years | 1310 | 37 | | (0.48) |
| Literacy of wife | | | | |
| Illiterate | 1687 | 51 | 1.74 | 3.50 |
| Literate | 1309 | 24 | | (0.06) |
| Religion | | | | |
| Hindu | 2566 | 66 | 1.08 | 0.03 |
| Muslim | 259 | 5 | | (0.87) |
| Age at consummation of marriage (ACM) | | | | |
| ⩽18 years | 2262 | 63 | 2.82 | 5.32 |
| >18 years | 734 | 12 | | (0.02) |
| Users of FP methods | | | | |
| Ever used | 580 | 12 | 0.75 | 1.42 |
| Never used | 2416 | 63 | | (0.35) |
| Total pregnancies | | | | |
| | 2996 | 75 | 1.11 | 4.07 |
| | | | | (0.04) |
| Fetal loss | | | | |
| No | 2128 | 49 | 1.10 | 0.10 |
| Yes | 868 | 26 | | (0.75) |
| Cervical erosion | | | | |
| Yes | 1357 | 38 | 1.21 | 0.47 |
| No | 1639 | 37 | | (0.49) |
| HSV | | | | |
| I + II | 2546 | 65 | 1.55 | 2.60 |
| No ab. | 450 | 10 | | (0.11) |
| HSV II | 1225 | 41 | 1.33 | 0.69 |
| I + No ab. | 1771 | 34 | | (0.41) |
| HSV[a] | | | | |
| II | 1225 | 41 | 1.38 | 2.44 |
| I | 1325 | 24 | | (0.12) |

[a]Sample size was different as women negative to HSV antibodies were excluded. Relative risk = exp (reg. coeff.).

**Table IV** Stepwise Cox regression analysis

| *Variables entered in the model* | *Log likelihood* | *Improved $\chi^2$* | *Global $\chi^2$ (P value)* | *d.f.* |
|---|---|---|---|---|
| ACM | −313.39 | | 5.32 (0.02) | 1 |
| ACM, total preg. | −312.25 | 2.28 | 7.85 (0.02) | 2 |
| ACM, total preg., literacy | −311.74 | 1.02 | 8.81 (0.03) | 3 |

### *Detection of HPV DNA sequences by in situ hybridisation*

The results of investigations for HPV revealed that of 63 progressive cases, 43 (68.3%) were found to be positive for HPV 16 and 18 DNA sequences, while out of 44 non-progressive cases, 12 (27.3%) were positive for HPV 16 and 18 DNA sequences. The difference was statistically significant ($P<0.001$) with a relative risk of 5.9 (95% CI = 2.5, 14.1).

### Discussion

The combined use of cytology and colposcopic monitoring provides a good opportunity to study factors which influence/determine the ultimate behaviour of dysplasias. The study was designed to follow up registered cases of dysplasia without any intervention. However, 103 (9.3%) cases were dropped during follow-up as they underwent hysterectomies for reasons other than progression to cancer. The hysterectomy rate was 3.4 per 100 women years. The hysterectomy rate for severe dysplasia was 8.69 per 100 women year, and for moderate and mild dysplasia the rates were 4.47 and 2.73 for 100 women years respectively. Hysterectomy rates for the controls were 1.1 per 100 women years (i.e. 30 cases (2.8%)), which were significantly lower than the hysterectomy rates for the matched dysplasia. The reason for differential hysterectomy rates remains unknown. However, this is not likely to affect the cumulative risk of progression as it has been shown that women undergoing hysterectomies for non-malignant conditions are probably at a lower risk of developing cancer of cervix than the general population (Miller, 1986).

As expected, the important determinant of risk of progression to malignancy was found to be the initial grade/severity of the preneoplastic changes. The rate of progression of severe dysplasia (42.0%) was considerably higher than progression of initially moderate dysplasia (24.3%) and initially mild dysplasia (4.9%) to cancer. The time lead bias alone may not explain this difference as sufficient time (132 months) was given to observe the behaviour or progression pattern of initially mild and moderate dysplasias. This indicates that mild and moderate dysplasia possibly constitutes a more heterogeneous category of cellular abnormalities with a variable potential of progression compared to sever dysplasia.

Progression to malignancy was found to be influenced by age at consummation of marriage (ACM). Women with consummation of marriage under 18 years of age had a 2.8-fold higher progression than those with ACM over 18 years. This may be possibly due to sexual insult to the younger cervix. We feel that earlier ACM increases the susceptibility of the cervix to the further action of carcinogens (Brinton & Fraument, 1986; Luthra *et al.*, 1987). That young tissue is more susceptible for the development of cancer has been shown for other cancers, notably hepatoma due to hepatitis B virus infection during the perinatal period. For oral cancer it has also been shown that initiation of tobacco chewing during adolescence increases the risk of oral cancer by 10-fold (Wahi, 1968).

It has been observed in Indian situations that Muslims have a lower incidence of cervical cancer than Hindus (Wahi *et al.*, 1972; Jussawala *et al.*, 1971). In this study, however, Muslim women with a significantly earlier age of consummation of marriage did not show significantly higher progression rates than Hindus. On the contrary, their progression rates were much lower, although not statistically significantly ($P > 0.05$). This could imply the presence of some protective factors operating in the Muslim women, such as better nutrition and genital hygiene. These factors, however, need to be evaluated in the Indian situation as malnutrition and poor genital hygiene are widely prevalent among Hindus, especially in the lower socio-economic class. A recent WHO report (WHO, 1986) considers genital hygiene to be an important factor in the Indian situation, especially when circumcision provides better penile hygiene.

Usage of family planning methods did not have any significant effect in modifying the progression rates to malignancy from dysplasia. It could be due to the fact that the large majority of subjects resorted to terminal methods of family planning, in both dysplasia and control groups.

In the present study women who revealed cervical erosion at the initial examination did not show a higher progression rate of malignancy than those without erosions. It has been shown earlier that diathermy coagulation of erosions leads to reduced risk of progression compared to no treatment (Vonka *et al.*, 1984*a*). In the present study, none of the erosions were treated. It could be that diathermy coagulation might have lowered the progression rates by destroying the transformation zone containing initiated cells. Thus erosion itself may not be an important risk factor for progression.

Three different analysis were made for studying HSV: (i) HSV I + II *vs* no antibody; (ii) HSV II *vs* HSV I + no antibody; (iii) HSV-II *vs* HSV-I. No significant increase in the progression rates was observed for dysplasia with any of the above combinations. Two prospective studies (Vonka *et al.*, 1984*b*; Adam *et al.*, 1985) employing a similar technology also failed to reveal any significant role of HSV in the process of cervical carcinogenesis.

At present the most seriously considered micro-organism is selective types of genital human papilloma virus (HPV). It has been reported that the risk of progression was considerably elevated in subjects with HPV infection (Howley, 1986).

Our investigations on HPV were carried out on the retrospective material only, employing a case–control design. Investigations for HPV could not be undertaken on the entire cohort as the significance of HPV as a possible aetiological agent was only realised globally during the later part of our study. For non-progressive cases, the investigations for HPV were carried out on the biopsies collected at the termination of the cohort. In progressive cases, the biopsy specimen obtained at the diagnosis of CIS was used for detection of HPV DNA sequences. With all these limitations, we obtained a very high odds ratio of 5.9 associated with progressive cases.

The study revealed that the 'sojourn time' for various grades of dysplasia did not differ according to various risk factors. This is inconsistent with the findings of others (Hakama, 1986).

In conclusion, the only risk modifiers identifiable in our study were initial grade of dysplasia, ACM (< 18 years) and the presence of HPV 16/18.

The authors wish to express their grateful thanks to Mr A.K. Prabhakar, Assistant Director (Bio-stat.), and Mr D.K. Shukla, Senior Research Officer (Programming), Indian Council of Medical Research, for their help in executing the BMDP package on their IBM PC.

### References

ADAM, E., KAUFMAN, R.H., ADDLER-STORTHZ, K. *et al.* (1985). A retrospective study of association of herpes simplex virus and human papillomavirus infection with cervical neoplasia in women exposed to diethyl still bestrol in utero. *Int. J. Cancer*, **35**, 19.

BRINTON, LA. & FRAUMENT, J.F. Jr (1986). Epidemiology of uterine cervical cancer. *J. Chron. Dis.*, **39**, 1065.

COX, D.R. (1972). Regression models and life tables (with discussion). *J.R. Stat. Soc. B*, **34**, 187.

HAKAMA, M. (1986). Cervical cancer. Risk groups for screening. In *Screening for Cancer of the Uterine Cervix*, Hakama, M., Miller, A.B. & Day, N.E. (eds). IARC: Lyon.

HOWLEY, P.M. (1986). On human papilloma viruses. *N. Engl. J. Med.*, **315**, 1089.

JUSSAWALA, D.J., DESHPANDE, V.A. & STANDFAST, S.J. (1971). Assessment of risk patterns in cancer of the cervix. A comparison between greater Bombay and Western Countries. *Int. J. Cancer*, **7**, 259.

KAPLAN, E.L. & MEIER, P. (1958). Non parametric estimation from incomplete observations. *J. Am. Stat. Assoc.*, **53**, 457.

LUTHRA, U.K., PRABHAKAR, A.K., SETH, P. & 5 others (1987). Natural history of precancerous and early cancerous lesions of the uterine cervix. *Acta Cytol.*, **31**, 226.

LUTHRA, U.K., PANDEY, R.N., PRABHAKAR, A.K. & RAVI, K. (1975). Prevalence and distribution of cancer of uterine cervix in an urban population. Proceedings of second Asian Conference on Cancer, Singapore.

MATHEWS, D.E. & FAREWELL, V.T. (1985). *Using and Understanding Medical Statistics*. Karger: New York.

MILLER, A.B. (1986). Evaluation of the impact of screening for cancer of the uterine cervix. In *Screening for Cancer of the Uterine Cervix*, Hakama, M., Miller, A.B. & Day, N.E. (eds). IARC: Lyon.

RIOTTON, G., CHIRTOPHERSON, W.M. & LURT, R. (1973). *Cytology of the Female Genital Tract: International Histological Classification of Tumours*, vol. 8. WHO: Geneva.

SETH, P., PRAKASH, S.S. & GOSH, D. (1978). Antibodies to HSV type I and II in patients with squamous cell carcinoma of uterine cervix in India. *Int. J. Cancer*, **22**, 708.

SHARMA, B.K., GUPTA, M.M., MURTHY, N.S. & 3 others (1985). Role of HSV antibodies in precancerous and cancerous lesions of the uterine cervix – a prospective study. *Ind. J. Med. Res.*, **85**, 282.

VONKA, N., KANKA, J., JELINCK, J. & 11 others (1984*a*). Prospective study on the relationship between cervical neoplasia and herpes simplex type 2 virus I. Epidemiological characteristics. *Int. J. Cancer*, **53**, 44.

VONKA, N., KANKA, J., HIRSCH, I. & 10 others (1984*b*). Prospective study on the relationship between cervical neoplasia and herpes simplex type 2 virus II. Herpes simplex type-2 antibody presence in sera taken at enrolment. *Int. J. Cancer*, **53**, 61.

WAHI, P.N., LUTHRA, U.K., MALI, S. & SHIMKIN, M.B. (1972). Prevalence and distribution of cancer of the uterine cervix in Agra District, India. *Cancer*, **30**, 710.

WAHI, P.N. (1968). The epidemiology of oral and oropharyngeal cancer. *Bull. WHO*, **38**, 495.

WHO (1986). Control of cancer of the cervix, a WHO meeting. *Bull. WHO*, **64**, 607.